# Imaging Assessment of Tumor Response in the Era of Immunotherapy

**DOI:** 10.3390/diagnostics11061041

**Published:** 2021-06-05

**Authors:** Jun Nakata, Kayako Isohashi, Yoshihiro Oka, Hiroko Nakajima, Soyoko Morimoto, Fumihiro Fujiki, Yusuke Oji, Akihiro Tsuboi, Atsushi Kumanogoh, Naoya Hashimoto, Jun Hatazawa, Haruo Sugiyama

**Affiliations:** 1 Department of Clinical Laboratory and Biomedical Sciences, Osaka University Graduate School of Medicine, Suita City 565-0871, Osaka, Japan; oji@sahs.med.osaka-u.ac.jp; 2 Department of Kansai BNCT Medical Center, Osaka Medical and Pharmaceutical University, Takatsuki City 596-8686, Osaka, Japan; kayakoisohashi@gmail.com; 3 Department of Cancer Stem Cell Biology, Osaka University Graduate School of Medicine, Suita City 565-0871, Osaka, Japan; yoshi@imed3.med.osaka-u.ac.jp; 4 Department of Immunopathology, WP1 Immunology Frontier Research Center, Osaka University, Suita City 565-0871, Osaka, Japan; kumanogo@imed3.med.osaka-u.ac.jp; 5 Department of Cancer Immunology, Osaka University Graduate School of Medicine, Suita City 565-0871, Osaka, Japan; hnakaji@cit.med.osaka-u.ac.jp (H.N.); fu-fuji@sahs.med.osaka-u.ac.jp (F.F.); sugiyama@sahs.med.osaka-u.ac.jp (H.S.); 6 Department of Cancer Immunotherapy, Osaka University Graduate School of Medicine, Suita City 565-0871, Osaka, Japan; soyoko@imed3.med.osaka-u.ac.jp (S.M.); tsuboi@cit.med.osaka-u.ac.jp (A.T.); 7 Department of Respiratory Medicine and Clinical Immunology, Osaka University Graduate School of Medicine, Suita City 565-0871, Osaka, Japan; 8 Integrated Frontier Research for Medical Science Division, Institute for Open and Transdisciplinary Research Initiatives (OTRI), Osaka University Graduate School of Medicine, Suita City 565-0871, Osaka, Japan; 9 Department of Neurosurgery, Kyoto Prefectural University of Medicine Graduate School of Medical Science, Kyoto City 602-8566, Kyoto, Japan; hashimotonaoya@me.com; 10 Department of Research Center for Nuclear Physics, Osaka University Graduate School of Medicine, Suita City 565-0871, Osaka, Japan; junhatazawa@gmail.com

**Keywords:** immunotherapy, response criteria, response assessment, false positive images

## Abstract

Assessment of tumor response during treatment is one of the most important purposes of imaging. Before the appearance of immunotherapy, response evaluation criteria in solid tumors (RECIST) and positron emission tomography response criteria in solid tumors (PERCIST) were, respectively, the established morphologic and metabolic response criteria, and cessation of treatment was recommended when progressive disease was detected according to these criteria. However, various types of immunotherapy have been developed over the past 20 years, which show novel false positive findings on images, as well as distinct response patterns from conventional therapies. Antitumor immune response itself causes 18F-fluorodeoxyglucose (FDG) uptake in tumor sites, known as “flare phenomenon”, so that positron emission tomography using FDG can no longer accurately identify remaining tumors. Furthermore, tumors often initially increase, followed by stability or decrease resulting from immunotherapy, which is called “pseudoprogression”, so that progressive disease cannot be confirmed by computed tomography or magnetic resonance imaging at a single time point. As a result, neither RECIST nor PERCIST can accurately predict the response to immunotherapy, and therefore several new response criteria fixed for immunotherapy have been proposed. However, these criteria are still controversial, and also require months for response confirmation. The establishment of optimal response criteria and the development of new imaging technologies other than FDG are therefore urgently needed. In this review, we summarize the false positive images and the revision of response criteria for each immunotherapy, in order to avoid discontinuation of a truly effective immunotherapy.

## 1. Imaging Assessment of Tumor Response before the Era of Immunotherapy

### 1.1. Imaging Techniques

Imaging techniques are used for diagnosis, staging, tumor response to treatment, and follow-up after treatment. Remarkable progress in imaging technology during the past half-century has improved the management of cancer patients, resulting in their better clinical results. Computed tomography (CT) and magnetic resonance imaging (MRI) are the most commonly used imaging techniques for solid cancers and malignant lymphomas (ML). They make it possible to detect the morphological changes seen in tumor masses and tumor metastases. In addition to such morphological imaging techniques, positron emission tomography (PET) using 18F-fluorodeoxyglucose (FDG) (FDG–PET) has come into use for the evaluation of the cell metabolism. Since tumor cells are hypermetabolic, which leads to their rapid proliferation, tumor involvements can be detected as lesions with FDG uptake by using FDG–PET. In addition, a combination of FDG–PET with CT (FDG–PET/CT) or MRI (FDG–PET/MRI) has been developed. With the FDG–PET/CT or FDG–PET/MRI, not only morphological but also metabolic aspects can be evaluated, so that small tumor involvements can be identified with higher sensitivity and specificity than is possible with CT alone [1,2]. As a result, not only CT and MRI, but also FDG–PET/CT and FDG–ET/MRI, are now routinely performed in oncotherapy.

### 1.2. Imaging Assessment of Tumor Response for Solid Tumors

One of the most important purposes of the use of imaging techniques is the assessment of tumor response during early treatment [3,4]. Since many kinds of anti-cancer drugs and treatment regimens are currently in use, prediction of the response to a particular therapy is important for changing it as soon as possible to other therapies for non-responders. The first published response criteria for the evaluation of tumor response for solid tumors were the World Health Organization (WHO) criteria in 1981 [5]. In these criteria, measurable lesions and the number of lesions to be assessed were not defined, and varied among research organizations. Since a common method for the assessment of tumor response was necessary in clinical trials for determining the effectivity of newly developing anti-cancer drugs, the response evaluation criteria in solid tumors (RECIST) were newly developed in 2000, and were subsequently updated to RECIST1.1 in 2009 [6,7]. These criteria defined the lesions to be assessed and the classification of response as complete response (CR), partial response (PR), stable disease (SD), or progressive disease (PD). In addition, unidimensional measurement in the RECIST criteria was less prone to bias than bidimensional measurement in the WHO criteria. As a result, many studies have found that RECIST1.1 was successful for predicting treatment outcomes [8,9,10,11]. However, this was only based on morphologic imaging. With small lesions, however, it is sometimes difficult to discriminate those involving tumors from non-tumor lesions—such as reactive changes or scars from old illnesses. Furthermore, tumor reduction with an effective therapy decided on the basis of morphological imaging may take several weeks, or even months [12]. It is therefore anticipated that with metabolic imaging the response to the therapy can be detected earlier, and with greater sensitivity. The first PET-based response criteria were published by the European Organization for Research and Treatment of Cancer (EORTC) in 1999 [13]. These criteria defined metabolic response in terms of changes in the maximum standardized uptake value (SUV) (ΔSUVmax) from baseline. A decrease of ≥15% in ΔSUVmax is defined as a partial metabolic response (PMR), a decrease of ≤15% to an increase of ≤25% as stable metabolic disease (SMD), and an increase of ≥25% as progressive metabolic disease (PMD). However, these criteria are specifically for metabolic assessment, and were not combined with morphological findings. Subsequently, new response criteria—the result of combining morphologic RECIST1.1 criteria and metabolic EORTC criteria—were introduced under the name of PET response criteria in solid tumors (PERCIST) in 2009 [14]. Many studies showed that the PERCIST1.0 were much effective than RECIST1.1 for the detection of early therapeutic response to chemotherapy [14,15,16]. This is how not only CT and MRI, but also FDG–PET/CT and FDG–PET/MRI, came into common use for the assessment of treatment response.

### 1.3. Imaging Assessment of Tumor Response for ML

Different criteria are used for the evaluation of response to ML [4]. The first standardized response criteria for ML were the International Workshop Criteria (IWC) in 1999 [4]. These criteria were based on morphological findings by CT, and the responses were classified into CR, unconfirmed CR (CRu), PR, SD, and PD, depending on the sum of tumor size of up to six of the largest lesions. All lymph nodes over 1.5 cm in size must have decreased to equal to or less than 1.5 cm in order to be classified as CR, and patients with any remaining lymph nodes over 1.5 cm are classified as CRu. Since lymphoma lesions easily form fibrosis or scars as a result of chemoradiotherapy, these criteria tended to underestimate the response to the therapy. The introduction of metabolic assessment by FDG–PET/CT solved this issue. Scars and fibrosis are metabolically negative, so FDG–PET/CT can differentiate actual residue of lymphoma cells from scars and fibrosis. In response, the International Workshop revised the IWC to IWC + PET criteria in 2007 [17]. CRu was deleted from IWC + PET criteria, and PET negative and positive patients who were classified as CR, CRu, PR, or SD by IWC were assessed as CR and PR by IWC + PET, respectively. As a result, interim FDG–PET/CT after two to four cycles of chemotherapy succeeded in predicting the outcome of chemotherapy [18,19].

## 2. Imaging Assessment of Treatment Response in the Era of Immunotherapy

During the past two decades, many types of immunotherapy–such as immunomodulatory drugs (IMiDs), monoclonal antibodies, cancer vaccines, immune checkpoint inhibitors (ICIs), and chimeric antigen receptor T cells (CAR-T)—have been developed. As a result, immunotherapy is now the fourth pillar of anticancer treatment, following operation, radiotherapy, and chemotherapy. However, such great success also raised new concerns regarding treatment assessment based on imaging. Anticancer immune reactions themselves affect imaging results, so that the response criteria that worked well before the era of immunotherapy sometimes lead to a misdiagnosis of the response to immunotherapy. The effect by immunotherapy on imaging starts with what is commonly called the “flare phenomenon”—this is an immune reaction characterized by FDG uptake on FDG–PET, and is sometimes accompanied by fever, elevation of white blood cell count, and transient morphologic increase within a few weeks. Afterwards, the stronger the new immunotherapy, the more apparent and more sustainable its effect on imaging becomes. Finally, immunotherapy may induce a morphologically detected increase equivalent to the conventional response criteria for PD, even during and after the effective treatment, which has become known as “pseudoprogression”. As a result, the conventional response criteria need to be fixed for tumor response to immunotherapy. In this section, we summarize how immunotherapy can affect the findings of imaging (summarized in Table 1), and how response criteria have been updated for each type of immunotherapy (summarized in Table 2), in their order of appearance.

### 2.1. IMiD Therapy

#### 2.1.1. Mechanism of Immune Response of IMiD Therapy

IMiDs is a genetic term for thalidomide and its analogues (lenalidomide, pomalidomide, iberdomide, and apremilast). Thalidomide had originally been approved as a sedative, hypnotic, or antiemetic, but was withdrawn because of severe teratogenic side effects. However, its antiproliferative, antiangiogenic, and immunomodulatory properties were discovered later, and IMiDs are now approved for malignancies such as myelodysplasia syndrome, multiple myeloma, and mantle cell lymphoma. The entire mechanism of IMiDs has not yet been clarified, but IMiDs can induce interleukin-2, interferon-γ, and tumor necrosis factor-α secretion from T cells, and therefore enhance the proliferation and antitumor activity of natural killer and T cells [20]. Furthermore, several reports have suggested that IMiDs weaken the immunosuppressive effects of regulatory T cells and myeloid-derived suppressor cells [21,22]. These immunomodulatory properties can alter the intratumoral immune status to an activated state.

#### 2.1.2. False Positive Images Resulting from IMiD Therapy

An immune reaction shown on images was first reported for chronic lymphocytic leukemia patients treated with lenalidomide [23]. In this study, 58% of the patients had experienced enlargement of the lymph nodes and/or spleen, which may be accompanied by a low-grade fever, rash, or increase in white blood cells. FDG–PET/CT detected it as an increase in FDG uptake in the lymph nodes, and the phenomenon was named “flare reaction”. Pathophysiologic findings revealed the infiltration of immune cells in tumor sites, indicating that the FDG uptake did not reflect tumor cells, but rather the antitumor immune reaction in tumor sites [24]. Afterwards, flare reactions caused by IMiDs were also reported for various kinds of ML [25,26,27]. This effect on imaging indicated that greater attention needed to be paid to tumor response assessment, but it was reported only in the first cycle of the therapy, and appeared to last only for a few weeks. It was therefore concluded that flare reaction could be differentiated from tumor progression on subsequent images.

### 2.2. Monoclonal Antibody Therapy

#### 2.2.1. Mechanism of Immune Response of Monoclonal Antibody Therapy

Antitumor monoclonal antibody therapy is a targeted therapy. This therapy does not directly kill tumor cells, but binds to target antigens on the surface of tumor cells, subsequently triggering an antitumor immune response such as complement-dependent cytotoxicity (CDC), antibody-dependent cell cytotoxicity (ADCC), or antibody-dependent cell-mediated phagocytosis (ADCP). Rituximab, which targets CD20 antigen, was the first therapeutic monoclonal antibody approved in oncology. The combination of rituximab with chemotherapy achieved a better response rate compared to that of chemotherapy alone, first for follicular lymphoma, followed by various kinds of B-cell ML, including diffuse large B-cell lymphoma [28,29]. As a result, the combination of rituximab and chemotherapy is now a standard first-line regimen for most types of B-cell ML. Following the success of rituximab, other monoclonal antibodies targeting antigens—such as CD30, CC chemokine ligands 4, human epidermal growth factor receptor 2, and vascular endothelial growth factor (VEGF)—were approved.

#### 2.2.2. False Positive Images Resulting from Monoclonal Antibody Therapy

Since high glucose turnover occurs in tumor sites as a result of antitumor immune responses including CDC, ADCC, and ADCP, increases in FDG uptake were detected there by FDG–PET/CT. This type of flare reaction was reported for ML patients treated with rituximab-containing regimens not only during the first treatment cycle, but also throughout the entire treatment and afterwards [30]. In addition, rituximab is generally used in combination with chemotherapy and steroids, so that few clinical symptoms accompany the immune flare. As a result, it is difficult to accurately distinguish FDG uptake resulting from a tumor from an immune flare. Consequently, the rate of false positive findings, namely FDG uptakes which were retrospectively judged to be non-tumor residue, reportedly increased more in patients treated with rituximab and chemotherapy than in those treated with chemotherapy alone [18,31,32]. Immune flare was also reported for brentuximab vedotin, which is a drug conjugated with anti-CD30 antibody and monomethyl auristatin E; it works mostly by delivery of monomethyl auristatin E into CD30-positive malignant lymphoma cells, which causes cell death, but can also lead immune reactions such as ADCP. Consistent with the mechanism, 4 out of 58 patients reportedly showed tumor flare response with an increase in FDG uptake after the first therapy cycle, which subsequently regressed [33]. Furthermore, one study evaluated the time course of tumor metabolism caused by ^90^Y-ibritumomab tiuxetan. An increase in metabolism was detected after the decrease in tumor activity in some lesions [34]. This increase was lower than the decrease in tumor activity, and continued for between 4 and 12 weeks after the administration of ^90^Y-ibritumomab tiuxetan. This temporary increase in FDG uptake may reflect an immune response induced by this monoclonal antibody.

Flare reaction was also reported for monoclonal antibodies used for solid tumors. Since these monoclonal antibodies have a shorter history than those for ML, and also because they were used after the second line of treatment, reports of immune flares have been very limited so far, and restricted only to bone lesions. Bone flare phenomena were firstly reported for patients with bone metastases of prostate or breast cancer treated with hormone therapy. Increased FDG uptake was thought to represent osteoblastic activity after an effective therapy [35,36]. This phenomenon had been relatively rare, but recently many cases have been reported, showing that bone flare phenomena are observed on FDG–PET/CT not only for patients with breast cancer, but also for those with non-small cell lung cancer (NSCLC), following the use of anti-VEGF antibodies such as bevacizumab and erlotinib [37,38,39]. The findings of these studies suggest that the addition of immune flare response to monoclonal antibodies on osteoblastic changes may lead to a higher incidence of false positive FDG uptake.

#### 2.2.3. Revision of Response Criteria Caused by Monoclonal Antibody Therapy

Although interim FDG–PET/CT after two to four cycles of chemotherapy had contributed to successful prediction of treatment outcome before the introduction of rituximab, prediction became less successful because of the false positivity caused by the addition of rituximab [40,41,42]. Relapse rates were still significantly higher for patients with FDG uptake than for those without FDG uptake on interim FDG–PET/CT, and negative predictive values still exceeded 80%. However, the specificity and positive predictive values of FDG uptake on interim FDG–PET/CT were decreased, ranging from 20 to 75% [43,44,45]. These results suggested that patients without FDG uptake were highly expected to be cured, but that treatment for patients with remaining FDG uptake should not always be changed. Based on these results, the 12th International Conference on Malignant Lymphoma in Lugano in 2013 concluded to not recommend changing treatment solely on the basis of findings by interim FDG–PET/CT after immunochemotherapy using rituximab [46] (Table 2). Since a visual assessment called the Deauville score is used for the assessment of FDG–PET/CT positivity in the criteria, quantitative measurements such as ΔSUVmax are expected for alternative prediction markers for treatment response. Indeed, some studies have demonstrated the superiority of quantitative measurements for interim FDG–PET/CT, but each of these studies was performed with a relatively small population, and cutoff values and the timing for imaging were discrepant [47,48]. Validations by larger studies are therefore necessary in order to reach a consensus.

### 2.3. Cancer Vaccine Therapy

#### 2.3.1. Mechanism of Immune Response of Cancer Vaccine Therapy

A mixture of cancer-cell-expressing antigens and adjuvants is percutaneously injected at intervals between one week and a few months in cancer vaccine therapy. The antigen-presenting cells phagocytose them and migrate to draining lymph nodes, and subsequently activate cancer-antigen-specific T cells. Thereafter, these cancer-antigen-targeting T cells migrate to and infiltrate tumor sites.

#### 2.3.2. False Positive Images Resulting from Cancer Vaccine Therapy

Acquired immune response induced by vaccine therapy can be expected to be stronger and last much longer than CDC, ADCC, and ADCP induced by monoclonal antibodies. Consistent with this expectation, FDG–PET/CT may detect such an adaptive immune response as increased FDG uptake in the case of infectious disease vaccines such as influenza vaccines [49,50] and, recently, COVID-19 vaccines [51]. Since these vaccines do not target antigen-expressing lesions, increased FDG uptake is mainly detected in vaccinated sites and their draining lymph nodes. Moreover, such increased FDG uptake was sometimes confusing in cancer patients as to whether a lesion with FDG uptake was due to cancer relapse or to reactive changes caused by infectious disease vaccines [52,53]. In the case of cancer vaccines, FDG uptake has been detected not only in draining lymph nodes and/or vaccinated sites, but also in tumor sites [54,55,56]. Furthermore, FDG uptake persisting in vaccinated sites for years even after the discontinuation of vaccination has been also reported in patients who showed favorable outcomes from cancer vaccine therapy [56]. Such strong immune responses also caused another type of false positive imaging effect known as “pseudoprogression”, which is especially evident in the field of neuro-oncology [57,58,59,60]. Pseudoprogression is an increase in the morphologic size of a tumor or the appearance of a new lesion followed by tumor regression without any changes in treatment. It was firstly recognized in glioblastoma patients who were treated with radiation in combination with the adjuvant temozolomide [61,62], and is reportedly related to enhanced inflammation, disruption of the blood–brain barrier, and increased vascular permeability [63]. Thereafter, occurrence of pseudoprogression came to be reported more frequently as a result of administration of immunotherapy treatments such as cancer vaccine therapy [58,60,64,65,66,67], and has been histopathologically observed as infiltration of CD8+ lymphocytes without any remaining tumor [68]. In addition to flare reaction, which makes metabolic response assessment difficult, pseudoprogression makes morphologic response assessment difficult. This means that, at that time, actual progression could be conclusively determined only by means of retrospective imaging. Response criteria specific to immunotherapy were therefore needed.

#### 2.3.3. Revision of Response Criteria Caused by Cancer Vaccine Therapy

For pseudoprogression resulting from temozolomide chemotherapy, response criteria known as the response assessment in neuro-oncology (RANO) criteria were proposed in 2010 [63]. According to these criteria, an increase in tumor volume of less than 25% assessed by contrast-enhanced T1-weighted MRI within the first 12 weeks after the completion of radiotherapy was classified as SD for patients whose clinical symptoms were stable or improved. However, the use of T-cell-based immunotherapies such as cancer vaccine therapy results in new patterns of response to and progression against tumors. One major characteristic response pattern is “durable response”, in which SD is maintained for long periods, and sometimes followed by a slow and steady decline in total tumor burden. In addition, pseudoprogression occurred with higher frequency and lasted for more than 12 weeks after the start of the immunotherapy [65,68,69]. These findings raised the crucial issue of misdiagnosis of patients who show early development of progressive imaging findings, but still derive therapeutic benefits from immunotherapy, as non-responders according to the RANO criteria. The same concerns were also generated by the use of ICIs, as described in a later section. Since there were few effective therapeutic interventions for gliomas, premature discontinuation of a truly effective immunotherapy because of pseudoprogression should have been avoided. Studies of patients with glioma treated with immunotherapies such as cancer vaccines have reported that pseudoprogressive radiographic findings mostly occurred within 6 months of treatment initiation, and that these early findings typically stabilized or improved within 3 months for most patients who ultimately derived clinical benefits [68,69,70]. Based on these findings, new immune RANO (iRANO) criteria were introduced, which added a 12-week pending rule to the RANO criteria, as a treatment assessment specifically for glioma treated with immunotherapy [66] (Table 2). According to the iRANO criteria, for patients who show imaging findings that meet PD by RANO criteria, including the appearance of new lesions, within 6 months of immunotherapy initiation, confirmation on follow-up imaging of actual PD 12 weeks later is needed before diagnosing patients as non-responsive to the immunotherapy.

#### 2.3.4. New Attempts to Use Imaging for Response Assessment in Cancer Vaccine Therapy

The fact that immune response itself could be detected with FDG–PET/CT made the assessment of true tumor progression difficult, but it also led to a new possibility for predicting treatment response. A recent report suggested that the SUVmax of vaccinated skin might be a predictive marker for evaluating the strength of immune response to cancer vaccine therapies [56]. Furthermore, a transient vaccination-induced increase in FDG uptake in the spleen could reflect the intensity of immune response to cancer vaccine therapy in a murine model, resulting in FDG–PET/CT becoming a possible new predictive marker for cancer vaccine therapy [71].

### 2.4. ICI Therapy

#### 2.4.1. Mechanism of Immune Response of ICI Therapy

ICIs are a kind of monoclonal antibody that does not bind antigens on cancer cells, but instead on intrinsic T cells; they block the breaking system of T cells, and thus reactivate exhausted intrinsic antitumor T cells. Ipilimumab, an anticytotoxic T-lymphocyte-associated antigen 4 (CTLA-4) antibody, was first ICI approved in 2011 for use against unresectable or metastatic melanomas. CTLA-4 is a homolog of CD28, and both are T-cell co-stimulatory molecules, and can bind to CD80 or CD86 on the surface of antigen-presenting cells. Since CTLA-4 has stronger affinity and avidity for this binding, CTLA-4 hampers T-cell activation via CD28. Ipilimumab can block such a regulatory mechanism. Thereafter, the anti-programmed death 1 (PD-1) antibodies nivolumab and pembrolizumab were approved for unresectable or metastatic melanomas. PD-1 is expressed on activated T cells, and the signaling via PD-1 results in T-cell exhaustion. Cancer cells evade immune attack by expressing programmed death ligand 1 (PD-L1), which binds to PD-1 on T cells. Anti-PD-1 antibody therapy can block such PD-1/PD-L1 binding, resulting in reactivation of the exhausted T cells. Following the success of anti-PD-1 antibodies for the treatment of melanoma, ICIs have now been approved for many kinds of tumors, such as NSCLC, renal cell carcinoma, squamous cell carcinoma of the head and neck, gastric cancer, and colorectal cancer.

#### 2.4.2. False Positive Images Resulting from ICI Therapy

Pseudoprogression, which had previously been observed as a result of temozolomide and cancer vaccine therapy in the field of neuro-oncology, was also recognized in melanoma patients during the ipilimumab treatment in 2009 [72]. In some patients, melanoma size initially increased after ipilimumab administration, but showed a delayed partial response when the treatment was continued. Anti-PD-1 antibody therapy was also found to induce pseudoprogression in melanoma patients, with frequencies reportedly ranging from 2.8% to 9.7% [72,73,74,75,76]. Thereafter, pseudoprogression was also detected in other solid tumors, including NSCLC and squamous cell carcinoma of the head and neck, with frequencies from 1.3 to 6.9% [77,78,79,80]. Pseudoprogression caused by ICIs sometimes lasted for more than 12 weeks [73], indicating that pseudoprogression resulting from ICIs was more pronounced, more frequent, and continued for longer periods than that from cancer vaccine therapy. This showed that modification of both the morphologic and metabolic response criteria for solid tumors was needed.

#### 2.4.3. Revision of Response Criteria Caused by ICI Therapy

##### Revision of Morphologic Response Criteria

**Figure 1:**
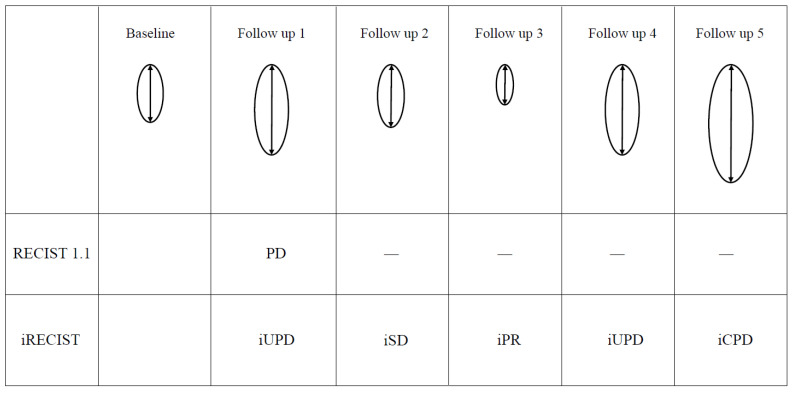
Illustration of a representative case for comparison between the RECIST and iRECIST criteria. Follow-up 1: Increase of ≥20% in the longest diameter of target lesion compared to the baseline is judged to be PD in the RECIST 1.1 criteria, and the treatment and tumor assessment are discontinued thereafter. On the other hand, the iRECIST criteria judge the size increase to be representative of iUPD, and the immunotherapy should be continued. Follow-up 2: Increase of the longest diameter compared to the baseline is <20%, which the iRECIST criteria judge to be iSD. The judgement of iUPD at follow-up 1 is reset at this timing. Follow-up 3: Decrease of the longest diameter compared to the baseline is >30%, which the iRECIST criteria judge to be iPR. Follow-up 4: Increase of ≥20% in the longest diameter of target lesion compared to the baseline is rejudged to be iUPD by the iRECIST criteria. Follow-up 5: Further increase of >5 mm compared to follow-up 4 is judged to be iCPD, and the immunotherapy should be discontinued at this stage.

Before the development of immunotherapy, RECIST1.1, which had been successfully validated in many clinical studies, was commonly used as the morphologic tumor response criteria, and cessation of the treatment was recommended when PD was detected. In addition, SD was usually transient and followed by PD in most cases, so only patients with CR or PR were considered to be responders for the treatment. However, clinical studies using ipilimumab for melanoma presented some distinct response patterns in patients with favorable survival, and in 2009 new response criteria for ipilimumab entitled irRC were proposed, with each response category’s names given the prefix of “ir”—thus, irCR, irPR, irSD, and irPD [73] (Table 2). Since durable response was often observed, irSD was evaluated as an effective time course for ipilimumab treatment. To avoid misinterpretation of pseudoprogression as PD, irPD required confirmation by a second imaging at least 4 weeks later if there was no rapid clinical exacerbation. Furthermore, the appearance of new lesions was sometimes observed in responders, so that the criteria specified that new lesions should not be considered to indicate irPD, but instead be added to the sum of target lesions. Since irRC criteria were based on WHO criteria, the definition of target lesions was 5 lesions per organ and up to 10 visceral and 5 cutaneous lesions and the measurements were bidimensional. Nishino et al. evaluated the revised irRC by using the same definition and measurement methods for target lesions as those of RECIST1.1, and proved that new criteria termed irRECIST were successful for predicting response to ipilimumab [81] (Table 2). Although these criteria proved to be effective for the prediction of response to ipilimumab in melanoma patients, accumulated data from the use of many types of ICIs for various types of tumors revealed that there still remained a pattern of misdiagnosis of pseudoprogression as PD, e.g., an initial increase in tumor size followed by continuing stability or only a minor decrease in size, which still corresponded to PD when compared to baseline. The RECIST Working Group therefore established new guideline response criteria in 2017, termed iRECIST, for all types of solid tumors treated with immunotherapy [82] (Table 2). In these criteria, each response category’s name was given the prefix of “i”, and a two-step confirmation system for PD was introduced in order to avoid misinterpretation. PD was divided into two categories—unconfirmed PD (iUPD), and confirmed PD (iCPD)—and when tumor progression was detected for the first time, patients were categorized as iUPD. Only when a ≥ 5 mm further increase occurred in the sum of measurements of target or new target lesions, a further increase occurred in non-target or new non-target lesions, or another new lesion appeared in the reassessment 4–8 weeks after the detection of iUPD, were the patients confirmed as iCPD. If the patients were categorized as iCR, iPR, or iSD after the detection of iUPD, the bar was reset and tumor progression for the next assessment was again categorized as iUPD (Figure 1). This “unconfirmed to confirmed” concept made it possible to avoid the discontinuation of the effective immunotherapy because of misinterpretation, but it required several months in order to accurately determine the treatment response.

##### Revision of Metabolic Response Criteria

Metabolic assessment combined with morphological assessment was found to be superior for early therapeutic assessment compared to morphological assessment alone, and the PERCIST criteria were reported to be effective for the prediction of treatment before the development of ICIs. However, the interpretation of FDG uptake requires attention, since enhanced FDG uptake can also represent activation of intratumoral T cells introduced by ICI therapy itself [83,84]. Several attempts have been made to determine the best assessment for interim FDG–PET/CT, and various response criteria have been proposed during the last decade. For example, in 2017, Cho et al. proposed new response criteria termed PECRIT [85] (Table 2). They compared four sets of response criteria—RECIST1.1, irRC, EORTC, and PERCIST—for prediction of optimal overall survival (OS) for 20 advanced melanoma patients treated with ICIs, and calculated the accuracy of these criteria as 75%, 70%, 70%, and 65%, respectively. RECIST1.1 was better than the others, and if SD patients were divided into two groups with a cutoff value of 15.5% for the peak SUV normalized by the lean body mass (SULpeak) of the hottest lesion, it could predict the eventual response to ipilimumab with 100% sensitivity and 93% specificity. As a result, the criteria introduced new classification terms consisting of “clinical benefit” and “no clinical benefit” in order to determine whether the treatment should be continued or not, and patients who were classified as CR or PR according to RECIST1.1, and patients who were classified as SD according to RECSIT1.1 with increases of <15.5% in the SULpeak of the hottest lesion, were classified as “clinical benefit”. On the other hand, another study in 2018 of 41 metastatic melanoma patients treated with ICIs reported that changes in SUV did not correlate with clinical response, but that the threshold of newly emerged FDG-avid lesions detected with post-therapy FDG–PET/CT imaging was a better predictive marker for the prediction of “clinical benefit”. As a result, new response criteria termed PERCIMT were proposed, and the criteria for “no clinical benefit” were defined as four or more new lesions of <1 cm, three or more new lesions of > 1 cm, or two or more new lesions of ≥1.5 cm in functional diameter in the criteria [86] (Table 2). On the other hand, there have been attempts to introduce and merge the concept of morphologic response assessment for immunotherapy into the PERCIST criteria. For example, Immunotherapy-mediated PERCIST5 (imPERCIST5), proposed by Ito et al. in 2019, was based on PERCIST combined with the irRC concept [87] (Table 2). For these criteria, new lesions were not determined as PMD, but instead were added to the sum of target lesions, and the SULpeaks of up to five lesions were used for metabolic assessment. imPERCIST5 was successful in predicting OS in 60 melanoma patients treated with ICIs. On the other hand, iPERCIST, which was based on PERCIST combined with iRECIST, was also proposed in 2019 [88] (Table 2). These criteria used a two-step confirmation system for PMD, and PMD was divided into unconfirmed PMD (UPMD) and confirmed PMD (CPMD)—the same as with iRECIST. As a result, 39% of NSCLS patients treated with ICIs were reclassified by iPERCIST compared to iRECIST, indicating that metabolic assessment provided additional prognostic information. In summary, a variety of FDG–PET/CT based response criteria have been proposed, but these criteria were based on results for a relatively small number of patients. Therefore, which algorithm is best for response assessment has not yet been decided, and might differ depending on tumor types or which ICI is used. Future studies are thus warranted.

#### 2.4.4. New Attempts to Use Imaging for Response Assessment in ICI Therapy

Further attempts have been made to use FDG–PET/CT for the prediction of response to ICIs. Anti-PD-1 antibody therapy was found to be clinically effective for only a limited number of patients, so the prediction of good responders was important for deciding the treatment strategy. Good responders were reportedly associated with PD-L1 expression and the frequencies of tumor-infiltrating lymphocytes [89]. Higher frequencies of tumor-infiltrating lymphocytes leads tumors to become inflamed, which can be detected as an increased FDG uptake on FDG–PET/CT. Consistent with this notion, several recent studies have demonstrated that SUVmax is associated with PD-L1 expression in lung cancer [90,91,92]. Furthermore, Takada et al. reported that the average SUVmax of the responders was significantly higher than that of non-responders for 89 NSCLC patients [93]. These findings suggest that baseline assessment of tumors by FDG–PET/CT could be a potential predictor of response to ICI therapy. Further studies are needed, however, in order to decide the relevant cutoff values and their accuracy for response prediction.

### 2.5. CAR-T Therapy

#### 2.5.1. Mechanism of Immune Response of CAR-T Therapy

CAR-T therapy is an adoptive transfer therapy of T cells that expresses engineered T-cell receptors fused with antibody-biding and T-cell-signaling domains. Transferred CAR-T cells bind tumor cells through antibody-biding domains, and then CAR-T cells activate and kill tumor cells. CD19-targeting CAR-T cells succeeded in showing clinical efficacy against B-cell acute lymphoblastic lymphoma, and were approved in 2017. Their approval was extended to relapsed or refractory diffuse large B-cell lymphoma in 2019.

#### 2.5.2. False Positive Images Resulting from CAR-T Therapy

Adoptively transferred CAR-T cells migrate and expand in tumor sites, and then a strong T-cell immune response can be expected to occur, and long-lasting CAR-T cells after adoptive transfer are needed for an effective outcome. From the principle of this therapy, immune flare and pseudoprogression should occur, as with other T-cell-based immunotherapies Indeed, several case studies have already reported that diffuse large B-cell lymphoma patients showed pseudoprogression following CD19-CAR-T therapy [94,95]. So far, however, the number of clinical experiments using CAR-T therapy has been very limited, and so future evaluations are needed in order to decide how to revise the response criteria for this therapy.

### 2.6. Summary of This Section

A variety of immunotherapies has been developed over the past 20 years, and they resulted in novel false positive findings and distinct response patterns different from those of conventional therapies. The stronger the immunotherapy became, the more difficult the assessment of the response became. At first, metabolic imaging at single time points could no longer solely confirm PD because of flare reactions. Next, morphologic imaging at single time points could also no longer confirm PD because of pseudoprogression. To overcome such misinterpretation on imaging, several new response criteria specifically for the assessment of immunotherapy have been proposed, most of which recommend confirmation of PD by secondary imaging at a later time point. These new criteria have succeeded in avoiding misinterpretation to some extent, but they also require several months in order to determine the assessment of treatment response.

## 3. New Imaging Techniques for the Future

Since FDG accumulation occurs not only in cancer cells, but also in the immune response itself, FDG–PET/CT now struggles to accurately assess remaining tumors in the era of immunotherapy. For this reason, new tracers that can distinguish tumor cells from immune response are under development, and one approach involves tumor-specific tracers. PET imaging by using ^11^C-labelled methionine (^11^C-MET), which can trace amino acid metabolism, was tested for patients with lung cancer and those with brain tumors in order to obtain cancer-cell specific imaging [96,97]. However, although ^11^C-MET accumulated cancers preferentially by large amino acid transporter (LAT), an elevated accumulation was also observed in normal tissue such as liver, pancreas, and pituitary gland tissues, which makes its use difficult in clinical settings. Thereafter, artificial amino acids such as ^18^F-fluoro-borono-phenylalanine (^18^F-FBPA) [98,99] and ^18^F-fluoro-α-methyl tyrosine (^18^F-FAMT) [100] were newly developed as cancer-specific tracers. These compounds are transported through LAT1, which is predominantly expressed on the cancer cell membrane of most major carcinomas in humans. Importantly, these PET tracers do not accumulate in inflammatory cells and normal tissue. Therefore, by combining FDG–PET/CT with LAT1–PET/CT, immune response activity could be assessed by determining the difference between FDG (tumor viability and immune response) and LAT1 tracer accumulation (tumor viability). The other approach to distinguish tumor cells from immune response is the labeling of adoptive immune cells with radioisotopes [101]. Indeed, ^111^In detectable by SPECT and ^89^Zr detectable by PET have already been tested for immune cell labeling in CAR-T therapy [102]. The development of these and other new imaging technologies can be expected to result in earlier and more accurate response assessment for immunotherapy.

## Figures and Tables

**Table 1:** False positive imaging and impact for response assessment for each immunotherapy.

	Commonly Used Drugs	Typical Tumor Types for Which False Positive Findings Were Reported.		Can Imaging Results at a Single Time Point Be Used for Decision to Change Therapy?	
		Flare Reaction (FDG Uptake)	Pseudoprogression	Morphologic Imagings (CT, MRI)	Metabolic Imagings (PET)
**IMiDs**	Thalidomide Lenalidomide Pomalidomide	CLL ML		Yes Should not be assessed during first cycle of therapy	Yes Should not be assessed during first cycle of therapy
**Monoclonal antibodies**	Anti-CD20 antibody Anti-HER2 antibody Anti-VEGF antibody	ML Bone metastasis		Yes	No Treatment not to be changed solely based on PET findings
**Cancer vaccines**	Peptide vaccine Dendritic cell vaccine	Ovarian cancer Pancreatic cancer	Glioma	No PD should be confirmed by second imagings	No Not enough validation
**ICIs**	Anti-CTLA4 antibody Anti-PD-1 antibody Anti-PD-L1 antibody	Melanoma NSCLC Neck cancer	Glioma Melanoma NSCLC Neck cancer	No PD should be confirmed by second imagings	No Not enough validation
**CAR-T cells**	CD19-CAR-T BCMA-CAR-T	ML	ML	No Not enough validation	No Not enough validation

FDG: fluorodeoxyglucose; CT: computed tomography; MRI: magnetic resonance imaging; PET: positron emission tomography; IMiDs: immunomodulatory imide drugs; ICIs: immune checkpoint inhibitors; CAR-T cells: chimeric antigen receptor T cells; HER2: human epidermal growth factor receptor 2; VEGF: vascular endothelial growth factor; CTLA4: cytotoxic T-lymphocyte-associated antigen 4; PD-1: programmed death 1; PD-L1: programmed death ligand 1; BCMA: B-cell maturation antigen; CLL: chronic lymphocytic leukemia; ML: malignant lymphoma; NSCLC: non-small cell lung cancer; PD: progressive disease.

**Table 2:** Response criteria for immunotherapy.

Years	Response Criteria (Tumor Type *)	Imaging for Assessment	Target Lesions	Management of New Lesions	Definition of PD (PMD)	Confirmation of PD (PMD)	Newly Introduced Features for Assessment of Immunotherapy
			Measurements				
2009	irRC	Morphologic	Up to 10 visceral and 5 cutaneous lesions: 5 per organ	Incorporate to the sum of the measurements.	Increase of > 25% in total tumor burden	Yes, at least 4 weeks later.	First criteria mentioning confirmation of PD, and also first criteria which do not classify new lesions as PD.
			Total tumor burden is defined as the sum of the products of two largest perpendicular diameters of all target lesions.				
2014	LUGANO 2014 guideline (lymphoma)	Morphologic and metabolic	Nodal: LDI > 1.5 cm Extra-nodal: LDI > 1.0 cm	Classified as PD	Morphologic: Increase of ≥50% in PPD & ≥0.5 cm for lesions ≤2 cm or ≥1.0 cm for lesions >2 cm Metabolic: Score of 3, 4, and 5 in 5PS with increased uptake compared to nadir.	No	Metabolic assessment has priority over morphologic assessment, but the therapy should not be changed solely by metabolic PD.
			Morphologic: Products of perpendicular diameters (PPD) Metabolic: 5PS				
2014	irRECIST	Morphologic	Up to 5 lesions: 2 per organ	Incorporate to the sum of the measurements.	Increase of >20% and >5 mm in total tumor burden	Yes, at least 4 weeks and up to 12 weeks later.	The same as irRC.
			Total tumor burden is defined as the sum of the largest diameters of all target lesions.				
2015	iRANO (Brain tumor)	Morphologic	Up to 5 lesions	Incorporate to the sum of the measurements.	Increase of ≥25% in total tumor burden	Yes, 12 weeks later, if PD is detected by 6 months from initiation of immunotherapy.	By 6 months from initiation of immunotherapy, 12 weeks pending is required for confirmation of PD.
			Total tumor burden is defined as the sum of the products of two largest perpendicular diameters of all target lesions.				
2017	iRECIST	Morphologic	Up to 5 lesions: 2 per organ	Classified as iUPD.	iUPD: (same as for RECIST1.1) 1. Increase of ≥20% or ≥5 mm in target lesion 2. Unequivocal progression in non-target lesions 3. Appearance of new lesions iCPD: 1. Further increase of ≥5 mm in target or new target lesions from last iUPD 2. Unequivocal further progression in non-target or new non-target lesions from last iUPD 3. Additional appearance of new lesions from last iUPD	Yes, at least 4 weeks and up to 8 weeks later.	Two-step confirmation system from iUPD to iCPD is introduced. Further progression from iUPD is needed for iCPD, and bar is reset when iUPD is followed by iCR, iPR or iSD.
			Total tumor burden is defined as the sum of the largest diameters of all target lesions.				
2017	PECRIT	Morphologic and metabolic	Up to 5 lesions: 2 per organ	Classified as PMD	(same as for RECIST1.1) 1. Increase of ≥20% or ≥5 mm in target lesion 2. Unequivocal progression in non-target lesions 3. Appearance of new lesions	No	Classification of response are as same as for RECIST1.1, but in addition new classification of “clinical benefit” is introduced to decide whether the therapy should be continued or not. Patients with PMD or SMD with SULpeak >15.5 are classified as “no clinical benefit”.
			Morphologic: Total tumor burden is defined as the sum of the largest diameters of all target lesions. Metabolic: SULpeak				
2018	PERCIMT	Metabolic	Numbers and functional diameter of new FDG-avid lesions.	Numbers and size of new lesions are used for definition of PMD.	1. Four or more new FDG-avid lesions (<1.0 cm in functional diameter) 2. Three or more new FDG-avid lesions (>1.0 cm in functional diameter) 3. Two or more new FDG-avid lesions (>1.5 cm in functional diameter))	No	The classification of “clinical benefit” is used as the same way as for PECRIT. Only patients with PMD are classified as “no clinical benefit”.
2019	imPERCIST5	Metabolic	Up to 5 lesions: 2 per organ	Incorporate to the sum of the measurements.	Increase of >30% in sum of SULpeak of target lesions.	No	Management of new lesions is as the same as for irRC.
			The sum of the SULpeak of all target lesions.				
2019	iPERCIST	Metabolic	Hottest single tumor lesion	Classified as UMPD	UMPD: 1. Increase of >30% in SULpeak 2. Unequivocal progression of non-targeting lesions 3. New appearance of FDG-avid lesions CMPD: 1. Further increase of >30% in SULpeak from last UMPD 2. Unequivocal further progression of non-targeting lesions from last UMPD 3. Additional new appearance of FDG-avid lesions from UMPD	Yes, at least 4 weeks and up to 8 weeks later.	Two-step confirmation system the same as used for iRECIST is introduced.
			SULpeak of the hottest lesion				

PD: progressive disease; PMD: progressive metabolic disease; LDI: longest transverse diameter; 5PS: Deauville five-point scales according to visual assessment; UPD: unconfirmed progressive disease; CPD: confirmed progressive disease; CR: complete response; PR: partial response; SD: stable disease; SULpeak: average standardized uptake value corrected by lean body mass within a 1-cm^3^ spheric volume of interest; UMPD: unconfirmed metabolic progressive disease; CMPD: confirmed metabolic progressive disease. * If response criteria are specific for some types of tumor, the tumor type is described in parentheses.

## Data Availability

Not applicable.
